# Effects of Methamphetamine Administration on Information Gathering during Probabilistic Reasoning in Healthy Humans

**DOI:** 10.1371/journal.pone.0102683

**Published:** 2014-07-25

**Authors:** Anna O. Ermakova, Pranathi Ramachandra, Philip R. Corlett, Paul C. Fletcher, Graham K. Murray

**Affiliations:** 1 Department of Psychiatry, University of Cambridge, Cambridge, United Kingdom; 2 Cambridgeshire and Peterborough NHS Foundation Trust, Cambridge, United Kingdom; 3 Department of Psychiatry, Yale University, New Haven, Connecticut, United States of America; 4 Behavioural and Clinical Neuroscience Institute, University of Cambridge, Cambridge, United Kingdom; Inserm, France

## Abstract

Jumping to conclusions (JTC) during probabilistic reasoning is a cognitive bias repeatedly demonstrated in people with schizophrenia and shown to be associated with delusions. Little is known about the neurochemical basis of probabilistic reasoning. We tested the hypothesis that catecholamines influence data gathering and probabilistic reasoning by administering intravenous methamphetamine, which is known to cause synaptic release of the catecholamines noradrenaline and dopamine, to healthy humans whilst they undertook a probabilistic inference task. Our study used a randomised, double-blind, cross-over design. Seventeen healthy volunteers on three visits were administered either placebo or methamphetamine or methamphetamine preceded by amisulpride. In all three conditions participants performed the “beads” task in which participants decide how much information to gather before making a probabilistic inference, and which measures the cognitive bias towards jumping to conclusions. Psychotic symptoms triggered by methamphetamine were assessed using Comprehensive Assessment of At-Risk Mental States (CAARMS). Methamphetamine induced mild psychotic symptoms, but there was no effect of drug administration on the number of draws to decision (DTD) on the beads task. DTD was a stable trait that was highly correlated within subjects across visits (intra-class correlation coefficients of 0.86 and 0.91 on two versions of the task). The less information was sampled in the placebo condition, the more psychotic-like symptoms the person had after the methamphetamine plus amisulpride condition (p = 0.028). Our results suggest that information gathering during probabilistic reasoning is a stable trait, not easily modified by dopaminergic or noradrenergic modulation.

## Introduction

Jumping to conclusions (JTC) is a cognitive bias consistently observed in people with schizophrenia and is associated with delusions (for reviews see [1]–[3]). When patients with delusions are asked to make decisions relying on probabilistic inference in the context of tasks in which they are able to select how much information to gather, patients have been shown to make decisions based on less information than healthy controls: hence the term “jumping to conclusions” [4], [5]. Little is known about the neurobiological basis of this bias. One putative neural substrate for probabilistic reasoning biases in psychosis is dopaminergic dysfunction, given evidence for dopamine function in decision-making [6] and strong evidence implicating dopamine dysfunction in psychosis [7]–[10].

Amphetamines act through releasing catecholamine neurotransmitters: mainly dopamine but also, to a lesser degree, noradrenaline and serotonin. Amphetamines act on plasma membrane monoamine transporters so that, instead of the uptake of catecholamine into the cell, the transport is reversed, releasing catecholamines into the synapse. They also affect the vesicular monoamine transporter-2 in such a way that dopamine is released into the cytoplasm of the nerve terminal. In addition, amphetamines inhibit monoamine oxidase, an enzyme responsible for the breakdown of catecholamines [reviewed in 11].

Amphetamines, in addition to their psychostimulant effect, have been shown to elicit psychotic symptoms, even after a single administration [12], [13]. Furthermore, in 50–70 percent of people with schizophrenia, amphetamine exacerbates the existing positive symptoms [14]. Nuclear medicine studies show that amphetamine induces higher release of dopamine in people with psychosis compared to healthy controls, and that the degree of dopamine release is linked to severity of induced symptoms [8], [15].

Although this is not the first study to investigate the effect of dopaminergic agents on JTC, it is the first one to include ratings of subclinical psychotic symptoms and their relation to decision-making. The aim of this study was to investigate whether the administration of methamphetamine would induce a JTC bias in a probabilistic inference data gathering task. Since amphetamine is known to induce psychotic-like symptoms and is used as a model of schizophrenia, we hypothesised that people would decrease the amount of information sampled under the effect of the methamphetamine, and this would correlate with the severity of the psychotic symptoms induced by the drug. Furthermore, we expect that the administration of the antipsychotic drug, amisulpride, would ‘rescue’ the performance and abolish psychotic symptoms. We were also interested in whether the baseline (placebo) level of information gathering would predict psychotic experiences after drug administration.

## Methods

### Participants and Pharmacological Conditions

The study was approved by the Cambridgeshire 2 National Health Service Research Ethics Committee. Eighteen healthy volunteers (11 of them men; mean age, 25.3 years [SD = 4.9]) without psychiatric or neurological disorders gave written informed consent and were included in the study. Participants attended on three visits, separated by at least 1 week. In one visit, they received an intravenous infusion over 10 minutes with a methamphetamine solution (0.3 mg/kg of body weight), approximately 3 hours before the probabilistic reasoning test, and a placebo tablet. In another visit, participants received the intravenous methamphetamine as described above, and they were given an amisulpride tablet (400 mg) approximately 1 hour before the infusion.

In the third visit, they received a saline infusion and a placebo tablet. The order of the visits was pseudorandomized for each participant in a counterbalanced manner. Participants, researchers who administered questionnaires and the probabilistic reasoning task, and psychiatrists who measured mental state were all blind to the pharmacological condition of the visit. One of the male participants was excluded because of an error during drug administration (suspected administration of amisulpride on two visits).

We chose a dose of 0.3 mg/kg of methamphetamine because it has previously been shown to be a well-tolerated dose that causes significant increases in striatal dopamine release [16]. The first peak of amisulpride plasma concentration is approximately one hour after oral administration [17], hence our decision to administer methamphetamine (or saline) at this time.

This dose of amisulpride is at the lower end of the ones used for treating acute psychotic episodes in clinical practice. Although there is individual variability between amisulpride plasma concentration and dose administered, 400 mg is usually considered a moderate dose, that leads to about 45–75% dopamine receptor occupancy in the striatum and 70–80% in temporal lobes and in the thalamus [18], [19]. In addition, from a pragmatic perspective, we had good evidence to predict that a single dose of 400 mg would be well tolerated, as it does not induce gross impairment of cognition or of sensory-motor coordination in healthy volunteers [17], and this dose usually does not induce acute dystonic reactions, which are distressing side-effects that amisulpride can cause at high doses.

### Rating Scales and Psychiatric Assessment

Before the test administration, participants were interviewed by an experienced psychiatrist who had passed the membership examination of the Royal College of Psychiatrists to measure the severity of any mild (prodromal) psychotic symptoms using the Comprehensive Assessment of At-Risk Mental States, sum of the subscales 1.1 Unusual Thought Content (such as attenuated bizarre delusions, attenuated Schneiderian first-rank symptoms), 1.2 Non-Bizzare Ideas (such as suspiciousness and paranoia), and 1.3 Perceptual Abnormalities [20], [21].

### Probabilistic reasoning task

Participants were administered the task as a part of a larger study, reported elsewhere [22]. Participants had to complete the probabilistic reasoning task on each of the three visits. Testing was roughly at the same time for all participants, approximately 3 hours after the methamphetamine or placebo injection. The instructions were the same for all three visits (see S1). The task was the classical “beads task” [4]. Participants were told that a sequence of beads would be drawn from one of two jars, each containing 1000 small beads (black beads and white beads were mixed in each jar). In one condition the ratio of black to white beads was approximately 85∶15 (see Supplementary Material for the exact sequences) and in another it was more difficult, 60∶40. There were four trials for each condition. After each bead was drawn, each participant had a choice of whether to see more beads or to guess which jar they came from. Participants could draw as many beads as they wanted before making the decision, leading to the outcome variable, “draws to decision” (DTD). The task was carried out as per the protocol described in [4], using actual beads in a jar, as opposed to a computerised simulation; in this classical version of the task, there is no explicit cost of information sampling, no feedback and no financial rewards.

Participants also completed two sequences of beads where they had to assess the probability that the bead had come from a particular jar. One sequence contained beads of predominantly one colour, while in the second sequence the first 10 beads support the hypothesis that the beads are being selected from one jar but the remaining 10 beads favour the opposite jar, similar to the study [5]. While the sequence of beads was presented, participants had to indicate their confidence that beads were being drawn from the black or white jar. All ratings were converted to scores ranging from 0 to 100 (indicating certainty in jar B). In both sequences we compared the mean confidence scores and in addition in the sequence where the jar probabilities switch we analysed ratings at draws 1, 10 (after the first 10 trials were consistent with jar B), and 20 (after the next 10 trials were consistent with jar A), similar to the work by Langdon et al. [23], [24].

The sequence of beads for each trial was predetermined (for an example sequence see supplementary information). The series presentations were pseudorandomised for each visit and participant, so that each the sequences were balanced across visit number and drug condition. The sequences we used were the same in different visits; however the trials were presented in different orders in a pseudo-randomised manner, and often the white/black beads were reversed – so that people would not remember or perceive it as the same sequence.

### Working memory

There is a possible involvement of the working memory impairments in the JTC bias [25], [26], and methamphetamine improves at least some types of memory [27]. To assess putatively confounding effects of the drug interventions on working memory, we administered both forward and backward digit span tests either shortly before or after the probabilistic reasoning task in a counterbalanced manner.

### Statistical analysis

The effects of methamphetamine on eliciting psychotic symptoms, draws to decision (DTD), confidence estimates and digit span forwards and backwards were assessed using repeated measures analysis of variance (ANOVA; SPSS 21, Chicago, Il). In the ANOVA of DTD we looked at condition (85∶15, 60∶40) by drug (placebo, methamphetamine, methamphetamine + amisulpride). Drug treatment order was initially included as between subject factor (to avoid sensitization effects – the possibility that response to methamphetamine on the second time would be greater than on the first) and dropped from the subsequent analysis when non-significant. We report two-tailed p-values, significant at p<0.05. When the assumption of sphericity was violated we applied Greenhouse-Geisser corrections. We also examined whether baseline (placebo) probabilistic reasoning function predicted severity of methamphetamine induced psychotic symptoms using Spearman's correlation coefficients. To check how correlated were the numbers of DTD across the 3 visits we calculated intraclass correlation coefficients with a two-way random effects model.

## Results

### Task performance (probabilistic reasoning)

**Figure 1:**
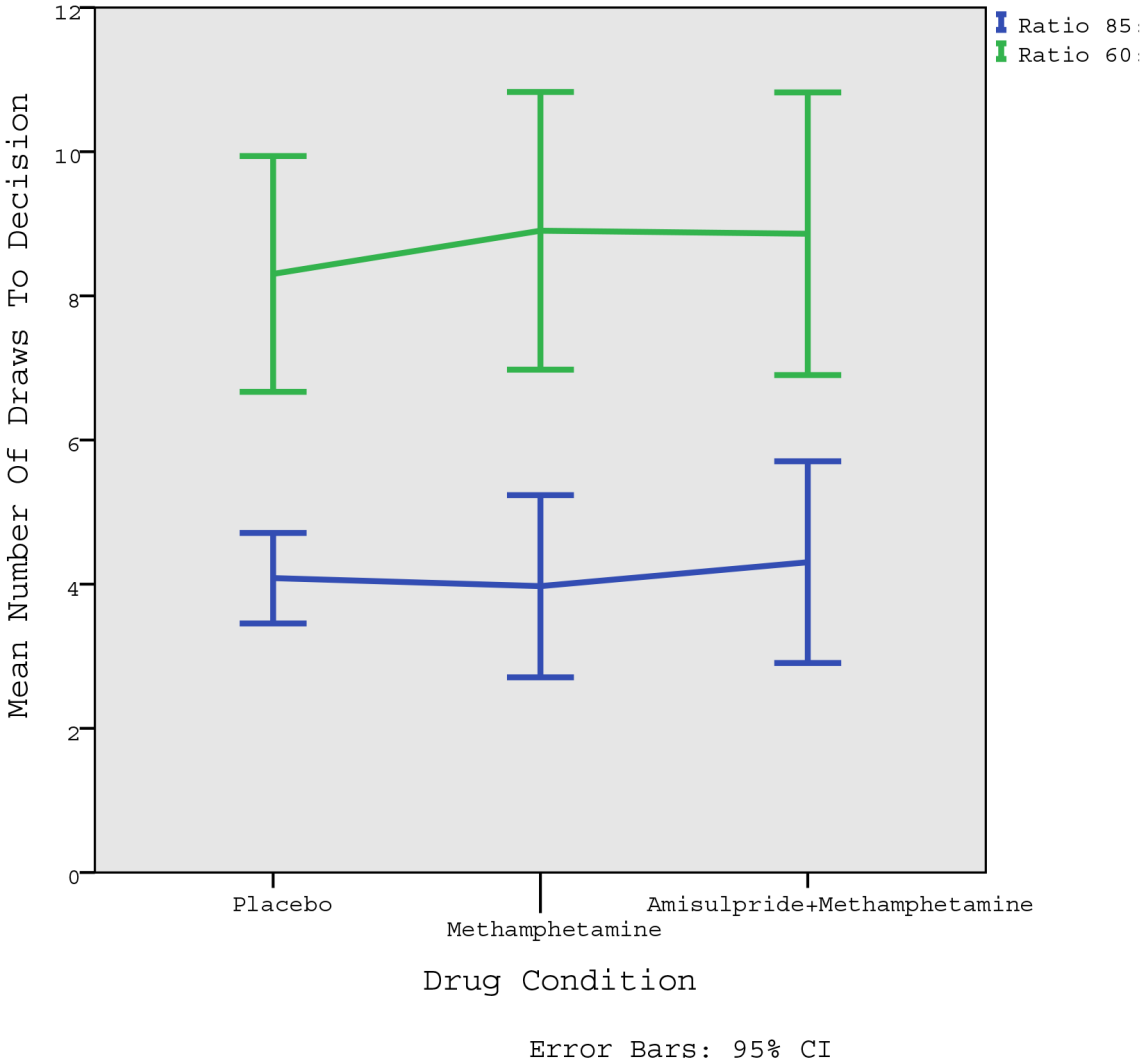
Mean number of draws to decision. Error bars are 95% confidence intervals.

**Figure 2:**
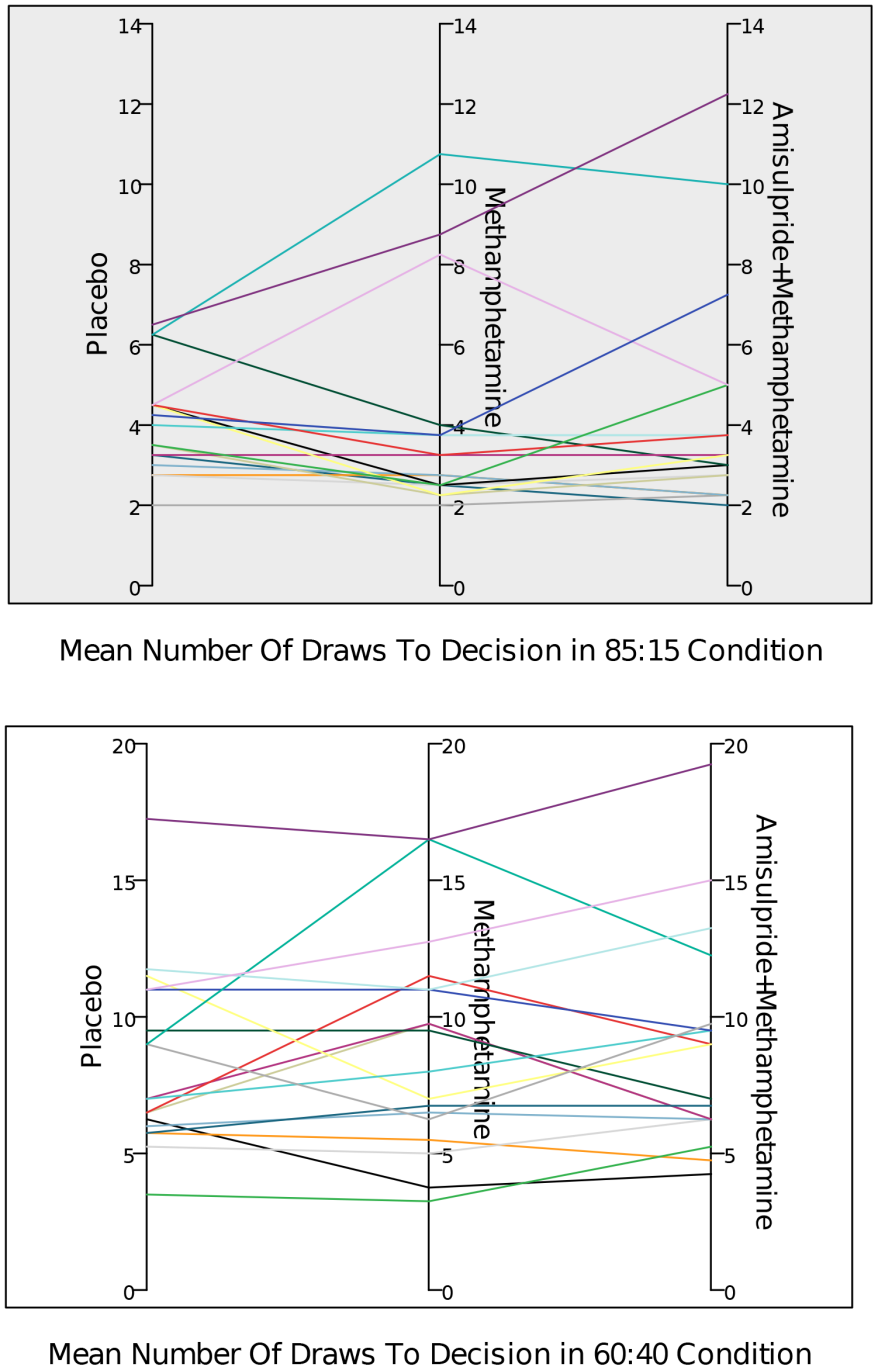
a,b. Draws to decisions of the individual participants in two experimental conditions during their 3 visits.

The mean number of DTD can be seen in Table 1 and Figure 1, while the individual variability can be observed in Figures 2a and 2b.

**Table 1:** Mean number of draws to decision.

DTD	Mean	Std. Deviation
Placebo 85∶15	4.08	1.26
Methamphetamine 85∶15	3.97	2.54
Amisulpride+methamphetamine 85∶15	4.31	2.81
Placebo 60∶40	8.31	3.29
Methamphetamine 60∶40	8.90	3.87
Amisulpride+methamphetamine 60∶40	8.86	3.94

Repeated measures ANOVA confirmed that there are no differences in the DTD between drug conditions (F(1.811) = 1.534, p = 0.630). However there are significant differences between ratios, i.e. whether participants looked at more beads in the more difficult, 60∶40 condition (F(1) = 83.97, p = 0.000). The drug*condition interaction was not significant (F(1.864) = 1.212, p = 0.472), indicating that changing the difficulty of the task had the same effect irrespective of the drug condition.

There was no effect of drug treatment order in either 85∶15 or 60∶40 conditions (F = 0.239, p = 0.788; F = 2.856, p = 0.072).

### Test-retest reliability

We wanted to examine the stability of the information gathering pattern across the three visits, i.e. whether participants who make hasty decisions would consistently do so on other visits and vice versa (Figure 2). To test this we calculated intraclass correlation coefficients (ICC). For a given subject, the information gathering pattern was stable across visits. For the 85∶15 ratio ICC = 0.857, F = 7.017, p<0.001. For the 60∶40 ratio ICC = 0.917, F = 12.092, p<0.001.

### Confidence estimates

**Figure 3:**
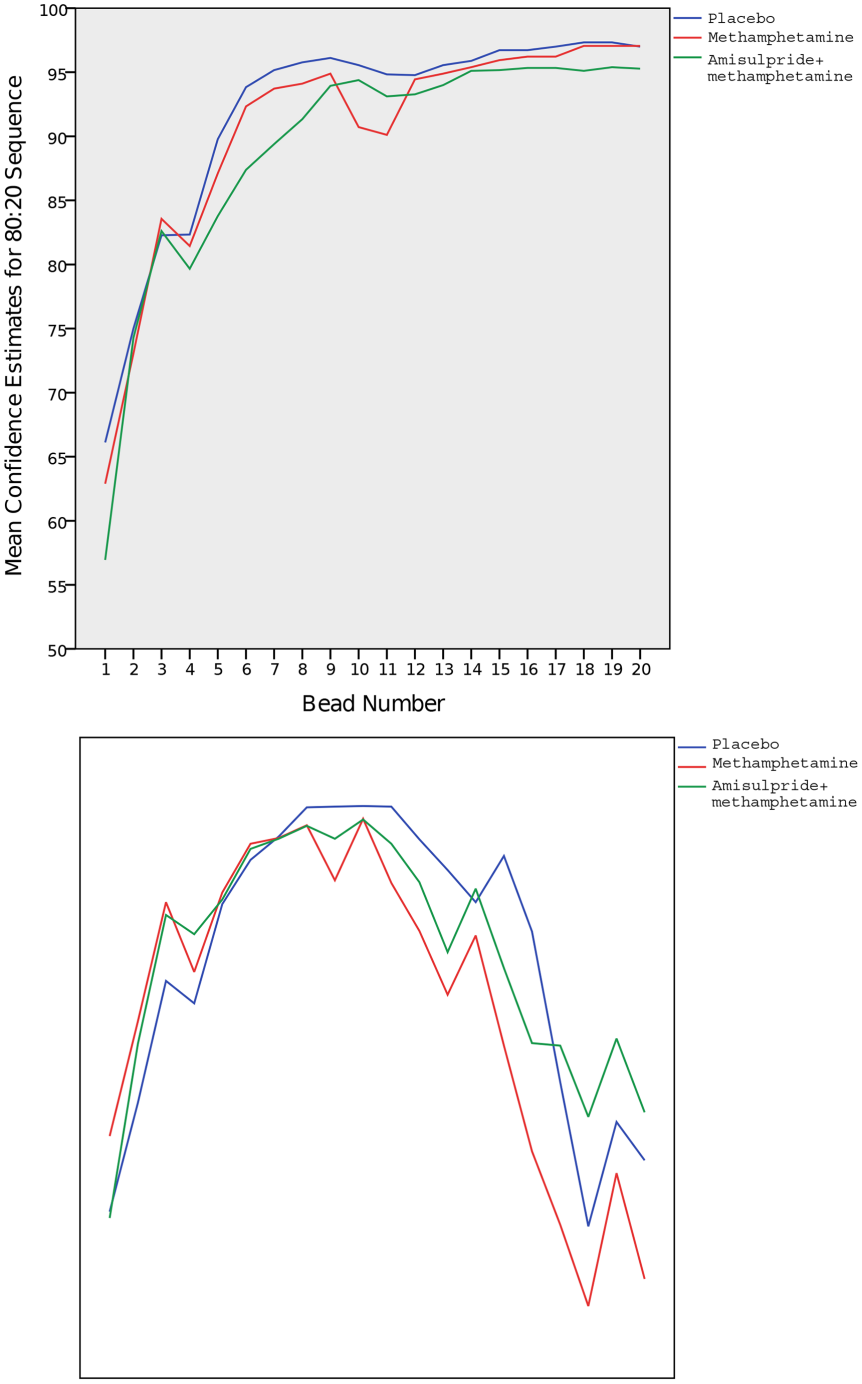
a,b. Mean confidence estimates for the two sequences in which participants were asked to rate the confidence of their decision. The red line represents the methamphetamine condition; blue represents the placebo condition, and green represents the combined amisulpride and methamphetamine condition.

Figure 3 illustrates the mean confidence estimates for both sequences in which participants were asked to rate their confidence in their belief that the beads were being drawn from a particular jar. Repeated measures ANOVA indicate that there is no significant drug effect in the mean confidence ratings for either sequence (F(1) = 1.878 p = 0.188; F(1) = 0.009 p = 0.925). There were no significant drug effects in the ratings for beads 1, 10 or 20 in the second confidence estimate sequence (F(1) = 0.346 p = 0.564; F(1) = 0.763 p = 0.395; F(1) = 0.018 p = 0.894).

### Psychotic symptoms

**Figure 4:**
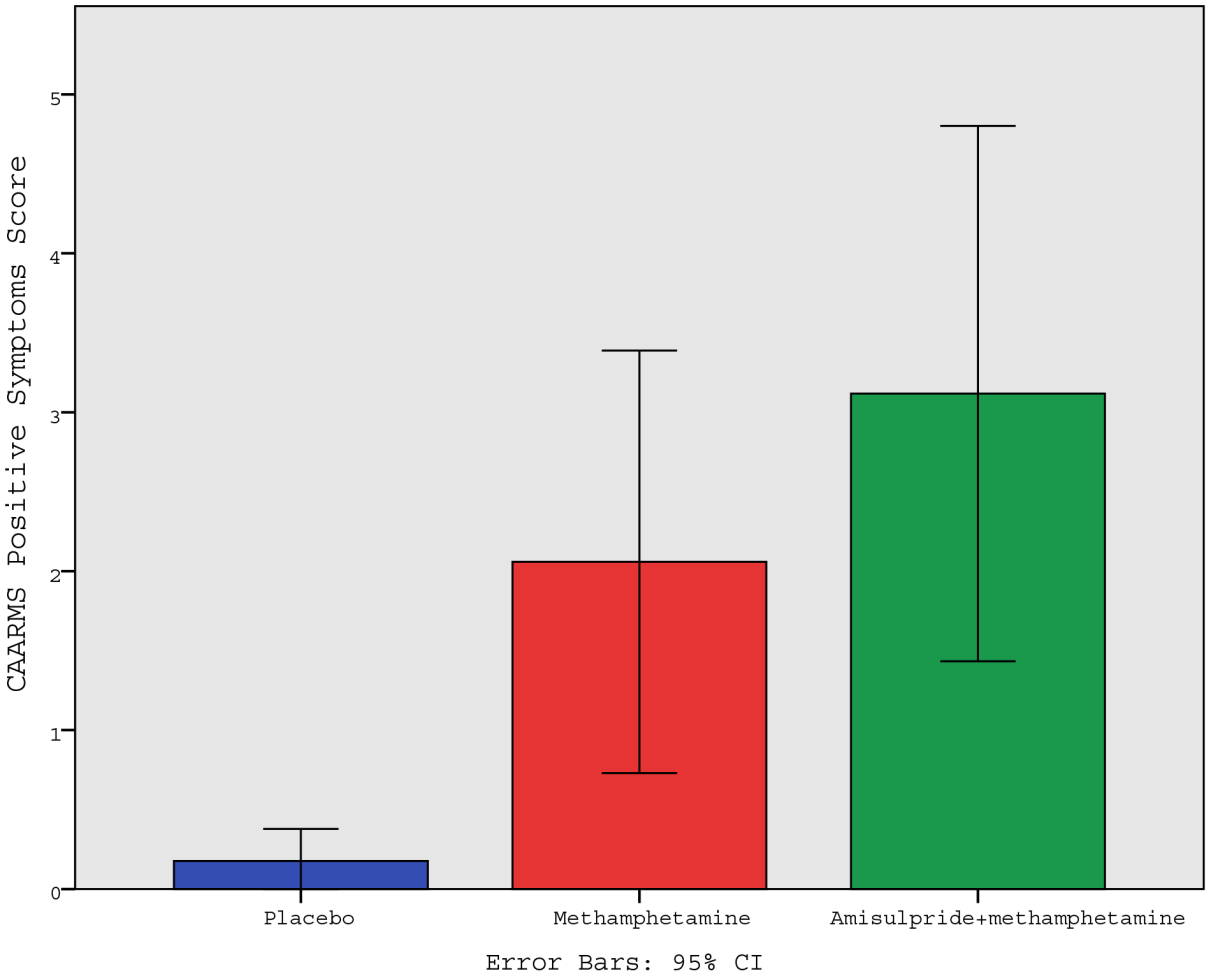
Main behavioural differences between drug conditions. Methamphetamine significantly induced psychotic symptoms in volunteers (p = 0.03, Bonferonni corrected), including with amisulpride pretreatment (p = 0.008 Bonferonni corrected). CAARMS = Comprehensive Assessment of At-Risk Mental States. Error bars are 95% confidence intervals.

Methamphetamine induced mild psychotic symptoms (as rated by the CAARMS), whether administered with placebo tablet or together with amisulpride (Figure 4) (placebo condition: score, 0.18 (SD = 0.39); methamphetamine condition: score, 1.94 (SD = 2.44); methamphetamine plus amisulpride condition: score, 3.12 ([SD = 3.28); main effect of drug condition: F = 8.859 (1.8), p = 0.002). Post-hoc comparisons for the condition differences are significant for the placebo versus methamphetamine (p = 0.032) and for the placebo versus methamphetamine plus amisulpride (p = 0.008), but not for the methamphetamine only versus methamphetamine plus amisulpride (p = 0.396).

### Working memory

Repeated measures ANOVA comparisons of the digit span forward and backward did not show any differences between the drug conditions (respectively F(1.60) = 1.184, p = 0.312; F(1.73) = 1.462, p = 0.247).

### Correlations between drug-induced symptoms and trait information gathering

To examine the relationships between methamphetamine-induced psychotic symptoms and the number of DTD we ran correlation analyses. We were interested in whether the placebo DTD measure represented a trait that could index vulnerability to methamphetamine induced psychosis. We used DTD measures from the placebo session in both 60∶40 and 85∶15 ratios and psychotic symptoms in both drug conditions. The only significant correlation was between the number of DTD in the 85∶15 condition in placebo and the positive symptoms on the CAARMS scale in the amisulpride + methamphetamine condition (ρ = −0.517, p = 0.028). To investigate this relationship further, we looked at the correlations with separate CAARMS subscales; the correlation was driven by the Unusual Thought Content subscale (ρ = −0.547, p = 0.019). This means that the more information was sampled in the placebo condition, the less psychotic symptoms the person had when taking both amisulpride and methamphetamine. The correlation between placebo DTD and severity of psychotic symptoms with methamphetamine only was not significant, although the direction of the association was the same but with a smaller effect size (ρ = −0.127, p = 0.616).

In the 60∶40 ratios correlations with CAARMS score under amisulpride +methamphetamine and methamphetamine alone were not significant (ρ = −0.216, p = 0.390; ρ = −0.077, p = 0.760 respectively).

## Discussion

Administration of methamphetamine (at least at our moderate dose) did not affect the amount of information gathered, or confidence estimates, during probabilistic reasoning, despite the fact that it induced mild psychotic-like symptoms in the healthy volunteers. Our data do not support the hypothesis that information gathering during probabilistic reasoning is subserved by catecholamine neurotransmission. A recent study examined the effects of single dose administration of haloperidol (a dopamine D2 receptor antagonist) and L-dopa (a dopamine precursor) on the probabilistic reasoning and the jumping to conclusions bias [28]. They found that neither drug influenced the number of draws to decisions or the probability of making the decision in a very similar task. We do note that one previous study has found that the amount of information gathered in a related paradigm (the CANTAB Information Sampling Task), was altered by administration of the dopamine D2/3 dopamine receptor agonist, pramipexole, in controls, though not in stimulant dependent individuals [29]. However, there are differences between the classical “beads” task as used in our study, and the IST, including that in the IST there are trials in which there is an explicit cost to data gathering, which could be relevant given the well established association between dopamine signalling and the reward system [30].

Whilst one interpretation of our results is that catecholamines do not subserve decision-making information sampling, we recognise that we need to consider several possible alternative explanations for our null result. Even though our cross-over design improves statistical power, given the relatively modest sample-size, the results we have could stem from the lack of power. We cannot rule out the possibility that a higher dose of methamphetamine might have had a stronger effect, but using higher doses of methamphetamine would present a higher risk of adverse effects. Furthermore, the dose (and sample size) was sufficient to demonstrate mental state effects (such as mild psychotic experiences), and, in another task in the same study, to alter behavioural and neural indices of reinforcement learning as reported by Bernacer and colleagues [22].

It is possible that methamphetamine may deleteriously influence decision-making information sampling, but that we failed to detect this because it also modulates other cognitive processes that act to obscure the effect on our outcome variable, number of draws to decision. For example, there is some evidence that other cognitive processes are associated with a JTC bias, including overconfidence in decisions under uncertainty [31], or reduced working memory capacity [3], [25]. If, for example, methamphetamine improved working memory but has a tendency to reduce information sampling during decision-making, the net result overall could be no-change in our measure of draws to decision. We attempted to account for this possibility by examining whether there was a drug effect on working memory as assessed by digit span forwards and backwards. We found no drug effect on working memory, or on confidence during probabilistic reasoning, but we concede the general principle that amphetamines can influence a variety of cognitive processes that could interact in complex ways to produce a net overall zero result.

Another possibility is that although catecholamines may influence information gathering, this is more likely to be modulated by chronic exposure than acute administration. Consistent with this account, in the related Information Sampling Task, Clark and colleagues showed that information gathering before making a decision is reduced in current and former amphetamine and opiate users [32]. Similarly, chronic cannabis users (but not ecstasy users) sampled significantly less information, and tolerated a lower level of certainty in their decision-making [33]. Nevertheless, studies of people who have been exposed to chronic drug use cannot differentiate between chronic effects of drug exposures and predisposing characteristics that lead certain individuals to develop chronic drug use.

It is possible that in healthy volunteers, information gathering during probabilistic reasoning may be a fairly stable “trait”. One line of evidence for this is that a JTC bias is observed in the relatives of the people with psychosis [34]. Other studies also confirm that this bias is not easily induced by drugs. Evans et al.[35] examined whether intravenous administration of the NMDA receptor antagonist ketamine modulated probabilistic inference in a version of the “beads” task. They were able to replicate the well-established finding that patients with schizophrenia drew less information during decision making compared to controls, but that ketamine had no effect on performance. The results of longitudinal studies in patients are somewhat mixed: in people with long-term illness the information gathering style (accessed either as DTD or JTC) remains stable [36], [37], while in people with early psychosis there has been found a decrease of information sampling in one study [38] and increase in the two others [39], [40]. The latter study had the longest follow up period (2 years) and found that less hasty decision makers were also less symptomatic. Menon and colleagues [41] had somewhat mixed findings, where DTD (but only on the emotionally salient version of the task) increased within two weeks of antipsychotic treatment and remained the same at week four. Baseline performance on the emotionally salient task predicted symptom improvement in response to antipsychotics. Our data show that healthy people have very stable information gathering styles (intraclass correlation coefficients of 0.86 and 0.91 on the two task conditions), and, when viewed with the existing literature, suggest that information gathering style during probabilistic inference is a stable trait.

Interestingly, the less information was sampled during our task at the baseline, placebo condition, the more psychotic symptoms (mainly unusual thought content) there were under the effects of combined amisulpride and methamphetamine. Whilst this could be a chance effect, it is also possible that the “jumping to conclusions” style of reasoning may be a cognitive trait that interacts with the hyperdopaminergic state engendered by methamphetamine to lead to the formation of psychotic symptoms. We have previously shown that baseline neurocognitive function can predict susceptibility to the psychotogenic effects of ketamine [42], [43]. Furthermore, Menon and colleagues found strong positive correlations in first episode schizophrenia between DTD in the emotional version of the JTC task with change in positive symptoms score both after 2 weeks and 4 weeks after treatment with antipsychotic medication: the more DTD people had on placebo, the more reduction in positive symptoms there was after treatment with antipsychotic medication. However, the degree to which DTD improved on treatment did not predict the response to treatment, which would be consistent with an account that the trait cognitive process could interact with the dopaminergic system to effect mental state changes [41]. In another study, published recently, So and colleagues investigated whether JTC bias at the baseline was connected to changes in the delusions dimensions after the initial 2 weeks of antipsychotic treatment [44]. They found that patients who showed a JTC bias at baseline showed little improvement in delusion associated distress and conviction after treatment, whereas patients who did not show JTC at baseline improved in these dimensions after treatment; this finding is consistent with the idea that the probabilistic reasoning style interacts with (or is a marker of a neural process that interacts with) the dopaminergic system to modulate psychopathology rather that directly being subserved by it.

The interpretation of the association between placebo DTD and drug-induced psychotic symptoms is complicated by the fact that the association was significant for symptoms induced by the combination of amisulpride and methamphetamine, and not significant for methamphetamine alone. One interpretation is that this is a chance effect: this suggests either a type I error in the amisulpride and methamphetamine condition or type II error in the methamphetamine alone condition. Although symptom severity was greater in the combination drug condition than with methamphetamine alone, this difference was not significant. Under this interpretation we should be cautious about drawing inferences about the difference between the two drug conditions. Another possibility is that there may truly be something pharmacologically specific about the combination of methamphetamine and amisulpride together that induces more symptoms and associates with trait probabilistic inference style: this would suggest contributions of dopamine D1 receptor agonism (as amisulpride does not block dopamine D1 receptors) or noradrenergic transmission (as amisulpride does not block noradrenergic actions) might be involved in psychotic symptom formation and relationship with trait information gathering style. Alternatively, as amisulpride in low doses has greater blockage of the presynaptic dopamine autoreceptors than postsynaptic receptors, thus facilitating dopamine release, it is possible that our dose of amisulpride had some autoreceptor-mediated pro-dopaminergic actions that contributed to psychotic symptom formation and interaction with trait information gathering style.

Administration of the dopamine D2 receptor antagonist antipsychotic amisulpride was not reflected in either task performance or reduction of the psychotic symptoms induced by methamphetamine. The dose we used, 400 mg is at the lower end of dose clinically used for treatment of psychosis, and, in this acute administration design, may have been insufficient to have a robust dopamine receptor antagonism effect.

## Conclusions

Administering methamphetamine to healthy volunteers, although inducing mild psychotic-like symptoms, did not result in hasty decision-making, which does not support the hypothesis that information gathering during decision-making is directly subserved by catecholamine neural systems.
